# A Multicomponent Vaccine Provides Immunity against Local and Systemic Infections by Group A Streptococcus across Serotypes

**DOI:** 10.1128/mBio.02600-19

**Published:** 2019-11-26

**Authors:** Shuai Bi, Meiyi Xu, Ya Zhou, Xinxin Xing, Adong Shen, Beinan Wang

**Affiliations:** a Key Laboratory of Pathogenic Microbiology and Immunology, Institute of Microbiology, Chinese Academy of Sciences, Beijing, China; b University of Chinese Academy of Sciences, Beijing, China; c Beijing Pediatric Research Institute, Beijing Children’s Hospital, National Center for Children’s Health, Beijing, China; University of Mississippi Medical Center

**Keywords:** B-cell responses, Th17, adaptive immunity, group A streptococcus, vaccines

## Abstract

Group A streptococcus (GAS) species are responsible for a broad spectrum of human diseases, ranging from superficial to invasive infections, and are associated with autoimmune disorders. There is no commercial vaccine against GAS. The clinical manifestations of GAS infection may be attributable to the large repertoire of virulence factors used selectively in different types of GAS disease. Here, we selected five molecules, highly conserved among GAS serotypes, and involved in different pathogenic mechanisms, as a multicomponent vaccine, 5CP. Intranasal (i.n.) immunization with 5CP protected mice against both mucosal and systemic GAS infection across serotypes; the protection lasted at least 6 months. Immunization of mice with 5CP constrained skin lesion development and accelerated lesion recovery. Flow cytometry and enzyme-linked immunosorbent assay analyses revealed that 5CP induced Th17 and antibody responses locally and systemically; however, the Th17 response induced by 5CP resolved more quickly than that to GAS when challenge bacteria were cleared, suggesting that 5CP is less likely to cause autoimmune responses. These findings support that immunization through the i.n. route targeting multiple nonredundant virulence factors can induce immunity against different types of GAS disease and represents an alternative strategy for GAS vaccine development, with favorable efficacy, coverage, duration, and safety.

## Abstract (precis)

GAS is among the most common human pathogens and causes a wide variety of diseases, likely more than any other microorganism. The diverse clinical manifestations of GAS may be attributable to its large repertoire of virulence factors that are selectively and synergistically involved in streptococcal pathogenesis. To date, GAS vaccines have not been successful due to multiple serotypes and postinfection sequelae associated with autoimmunity. In this study, five conserved virulence factors that are involved in GAS pathogenesis were used as a combined vaccine. Intranasal immunization with this vaccine induced humoral and cellular immune responses across GAS serotypes and protected against mucosal, systemic, and skin infections. The significance of this work is to demonstrate that the efficacy of GAS vaccines can be achieved by including multiple nonredundant critical virulence factors and inducing local and systemic immunity. The strategy also provides valuable insights for vaccine development against other pathogens.

## INTRODUCTION

Group A streptococcus (GAS) species are leading human bacterial pathogens with diverse clinical manifestations, ranging from mild skin infections, such as impetigo, to serious conditions, such as necrotizing fasciitis, streptococcal toxic shock syndrome, and scarlet fever. Repeated GAS infections are linked to autoimmune sequelae, including acute glomerulonephritis and rheumatic heart disease (1, 2); however, commercial vaccines against this pathogen are not yet available. The major obstacles hindering GAS vaccine development are serotype diversity and the potential for autoimmune responses related to these pathogens (3).

The upper respiratory tract (URT) mucosa is a common site of GAS colonization. Parenteral immunization does not protect against mucosal infection (4), because it fails to induce mucosal IgA and a dominant Th17 response, both of which are required for efficient GAS clearance (5–7). We previously demonstrated that intranasal (i.n.) immunization elicits both mucosal and systemic immune responses and protects against colonization of the URT mucosa and systemic infection (6), suggesting that i.n. immunization may protect against different clinical manifestations of GAS infection.

The diverse clinical manifestations of GAS are attributed to their large arsenal of virulence factors (2), which can be expressed at different stages of infection, as needed. Several GAS virulence factors induce protective immunity when used as single vaccine candidates (2, 8); however, single-antigen vaccines are unlikely to induce an immune response to other crucial virulence factors and may be inefficient against clinical isolates lacking the target antigen (3, 9, 10). We previously reported that a single conserved molecule (sortase A [SrtA]) induces serotype-independent protection against GAS in the URT mucosa (5). In addition, including one additional conserved virulence factor, streptococcal C5a peptidase (SCPA), into the SrtA vaccine increases the efficacy of local protection, as well as protecting mice systemically (6); however, the systemic protection effects in mice immunized with SrtA/SCPA are less than those in mice that have experienced GAS infection. Recently, more studies have demonstrated that vaccines containing multiple antigens confer efficient protection in some challenge models (11–14). We hypothesized that vaccination through the natural GAS infection route, with a set of conserved virulence factors involved in different bacterial pathogenic mechanisms, would ensure the efficacy and coverage of a GAS vaccine.

The goal of the present study was to evaluate the efficacy of immunity induced by a combined multiple-component vaccine, 5CP, in protection against different types of GAS infection and the associated immune responses. We demonstrate that i.n. immunization with 5CP protects against mucosal and systemic infection, independent of GAS M serotypes. In addition, 5CP-induced immunity constrained skin lesion development, promoted lesion recovery, and provided protection in a subcutaneous invasive disease model. These findings suggested that i.n. immunization with multiple virulence factors may be an optimal strategy for GAS vaccine development.

## RESULTS

### An antibody response was induced by i.n. immunization with 5CP.

**FIG 1:**
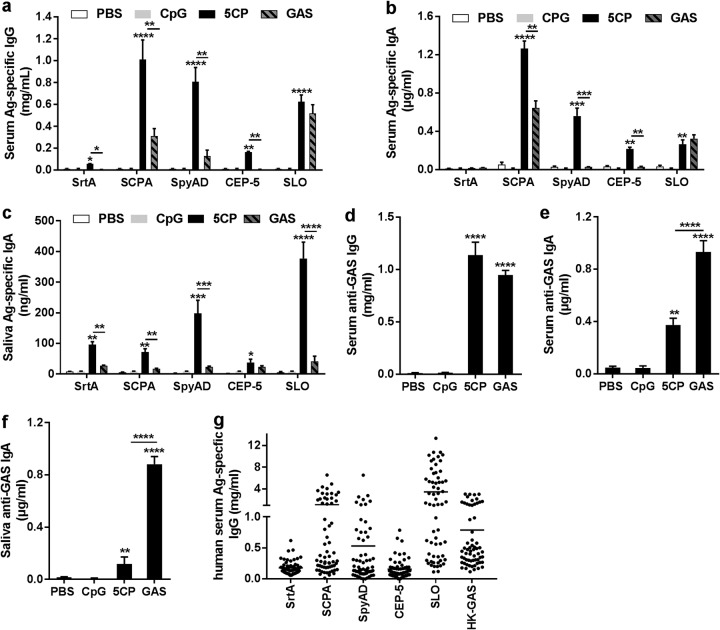
Antibody responses induced by 5CP. (a to f) Mice were intranasally immunized with 5CP or infected with group A streptococcus (GAS). Two weeks after the last immunization or infection, antigen (Ag)-specific antibodies were measured in serum and mouth wash samples by enzyme-linked immunosorbent assay (ELISA). (a to c) Serum IgG (a), serum IgA (b), and IgA in mouth wash (c), directed to each 5CP antigen. (d to f) Serum IgG (d), serum IgA (e), and IgA in mouth wash (f), directed to whole-cell GAS. All data are presented as means plus standard errors of the means (SEM) (error bars) from two or three independent experiments (*n* = 9 to 12). Statistical significance was determined by one-way ANOVA with Tukey’s posttest and indicated by asterisks as follows: ****, *P* < 0.0001; ***, *P* < 0.001; **, *P* < 0.01; *, *P* < 0.05. (g) Antigen-specific human serum IgG responses to the indicated 5CP antigens or heat-killed (HK) GAS (*n* = 62).

Five conserved GAS virulence factors were formulated as a multicomponent vaccine, designated 5CP, including SrtA (5), SCPA (15), the adhesion and division protein (SpyAD) (16), a fragment of SpyCEP (CEP-5) (17, 18), and streptolysin O (SLO) (19). The purified recombinant proteins were assessed by SDS-PAGE (see Fig. S1 in the supplemental material). Mice were i.n. immunized with 5CP, using CpG-oligodeoxynucleotides (CpG) as an adjuvant. Control groups were given phosphate-buffered saline (PBS) or CpG in PBS or infected with a low dose of live GAS M1 (strain 90-226). Serum and mucosal antibodies directed against each 5CP antigen were induced in 5CP-immunized mice (Fig. 1a, b, and c). Strong serum antibody responses to SCPA and SpyAD, and mucosal response to IgA to SLO, were observed. Most antibody responses to individual 5CP components were higher than those induced in infection-experienced mice, indicating that targeted antibody responses are more proficiently induced by 5CP than by whole bacteria.

10.1128/mBio.02600-19.1 FIG S1 Purified recombinant proteins were analyzed by SDS-PAGE. Lane 1, molecular weight marker; lane 2, SLO; lane 3, CEP-5; lane 4, SpyAD; lane 5, SCPA; lane 6, SrtA. Download FIG S1, PDF file, 0.09 MB. Copyright © 2019 Bi et al. 2019 Bi et al. This content is distributed under the terms of the Creative Commons Attribution 4.0 International license.

Enzyme-linked immunosorbent assay (ELISA) analyses were also performed to determine whether these antibodies could recognize GAS. Serum IgG from mice immunized with 5CP displayed levels of bacterial cell binding similar to those from GAS infection-experienced mice (Fig. 1d). The IgA in sera and mouth wash samples from the 5CP group also recognized the bacteria, but with lower binding capacity, relative to those from infection-experienced mice (Fig. 1e and f), indicating that 5CP antibodies can recognize target molecules natively situated on the bacterial surface. To determine the antigenicity of 5CP in humans, serum samples from 62 children (aged 5 to 15 years) were examined. As shown in Fig. 1g, IgG responses to each 5CP antigen were detected with relatively higher levels of responses to SCPA, SpyAD, and SLO as seen in mouse IgG, indicating that 5CP is antigenic in humans.

### Th17 responses were induced by i.n. immunization with 5CP.

T helper type 17 (Th17) cells can function as B cell helpers. They induce the formation of germinal centers and invoke strong proliferative responses in B cells, leading to pronounced antibody production (20, 21). In addition, interleukin 17 (IL-17), produced by Th17 cells, promotes neutrophil activation (22). We previously reported that clearance of streptococcal infection at mucosal sites is impaired in Th17-deficient mice (5, 7), demonstrating the important role of Th17 cells in anti-GAS immunity.

**FIG 2:**
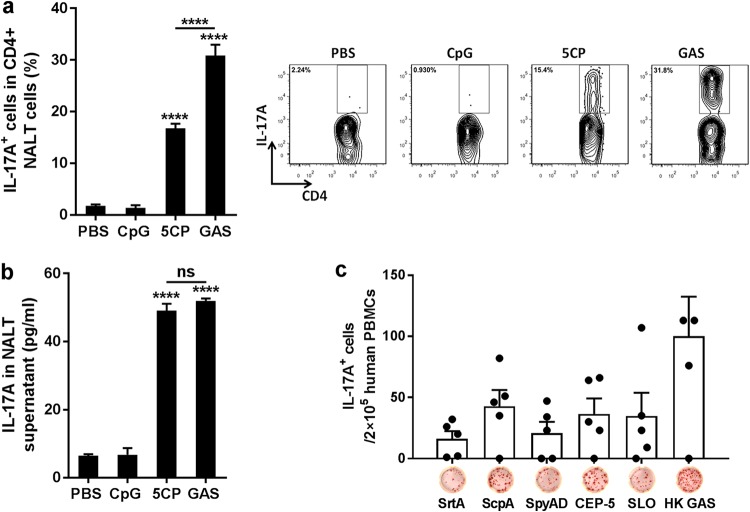
T cell responses induced by intranasal (i.n.) immunization with 5CP. (a and b) Mice were immunized as described in the legend to Fig. 1. Two weeks after the last immunization, mice were challenged i.n. with GAS. Five days after challenge, nasal-associated lymphoid tissue (NALT) samples were collected. (a) IL-17A^+^ CD4^+^ cells in NALT determined by flow cytometry. (b) IL-17A in supernatants of NALT cell homogenates measured by ELISA. Data are presented as the means plus SEM from two independent experiments (*n* = 8). Statistical significance was determined by one-way ANOVA with Tukey’s posttest and indicated as follows: ****, *P* < 0.0001; ns, not significant. (c) Human peripheral blood mononuclear cells (PBMCs) were stimulated with the indicated 5CP components or HK GAS, and IL-17A-secreting cells were measured by ELISPOT assay (*n* = 5).

Flow cytometry analyses of Th17 responses to 5CP revealed that CD4^+^ IL-17A^+^ cells were increased in nasal-associated lymphoid tissue (NALT) from 5CP-immunized mice, 5 days after the last immunization, with lower levels than those observed for GAS infection-experienced mice (Fig. 2a); however, levels of soluble IL-17A in the supernatants of homogenized NALT were comparable to those from infection-experienced mice (Fig. 2b). T cell enzyme-linked immunosorbent spot (ELISPOT) assays using human peripheral blood mononuclear cells (PBMCs) showed that antigen-specific IL-17A-positive (IL-17A^+^) T cells were detected in three or four of the five human PBMC samples (Fig. 2c), suggesting that 5CP can induce T cell responses and contains T cell epitopes in humans.

### Immunization with 5CP provided cross-serotype protection against GAS mucosal and systemic infection.

**FIG 3:**
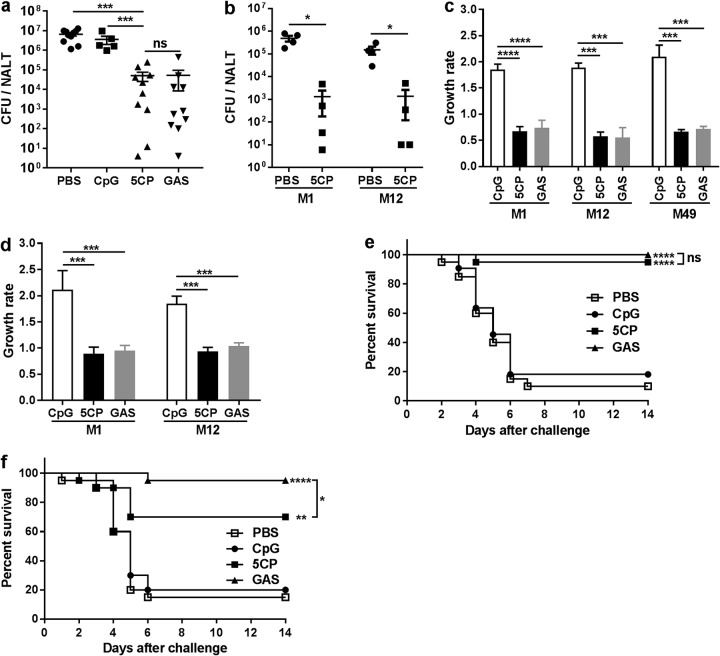
Immunization with 5CP through the i.n. route provided cross-serotype protection against GAS mucosal and systemic infection. Mice were immunized as described in the legend to Fig. 1. (a) Two weeks after the last immunization, mice were i.n. challenged with GAS M1. Colony-forming units (CFU) in NALT were determined 24 h after challenge. Data are from two independent experiments and presented as means ± SEM (*n* = 5 to 10). Statistical significance was determined by one-way ANOVA with Tukey’s posttest and indicated as follows: ***, *P* < 0.001; ns, not significant. (b) Immunized mice were challenged with GAS M1 or GAS M12. CFU in NALT were determined 24 h after challenge (*n* = 5). Statistical significance was determined by unpaired Mann-Whitney *U* nonparametric *t* tests and indicated as follows: *, *P* < 0.05. (c) Bacteria (GAS M1, M12, or M49) were coincubated with whole blood from immunized (5CP) or GAS-infected mice for 2 h, and bacterial growth rate was determined. (d) Bacteria (GAS M1 or M12) were coincubated with differentiated HL-60 cells in the presence of serum from immunized (5CP) or infected (GAS) mice for 1.5 h, and the bacterial growth rate was determined. Data are from two independent experiments and presented as means plus SEM (*n* = 8). Statistical significance was determined by one-way ANOVA with Tukey’s posttest and indicated as follows: ****, *P* < 0.0001; ***, *P* < 0.001. (e and f) Two weeks after the last immunization, mice were i.n. challenged with a lethal dose of GAS M1 (e) or M49 (f). Data are from two independent experiments (*n* = 20 or 21). Curves were compared using the log rank test for significance, and statistical significance was indicated as follows: ****, *P* < 0.0001; **, *P* < 0.01; *, *P* < 0.05; ns, not significant.

The efficacy of 5CP in protecting against GAS mucosal infection was first evaluated using an i.n. challenge mouse model. 5CP-immunized mice were challenged i.n. with GAS serotype M1, and CFU in NALT were determined 24 h later. We found that CFU were reduced to 5 × 10^4^ in 5CP-immunized mice compared with 5 × 10^6^ to 8 × 10^6^ CFU in PBS- or CpG-immunized mice, and similar to GAS infection-experienced mice (Fig. 3a), indicating promising protection in our models. As 5CP contains highly conserved streptococcal antigens, broad protection was expected. A group of 5CP-immunized mice was i.n. challenged with GAS serotype M12, which is the most frequently isolated serotype from cases with scarlet fever (23, 24). Similar to the protection against M1 serotype, CFU in 5CP-immunized mice were efficiently reduced after M12 challenge (Fig. 3b), indicating that 5CP-induced immunity can efficiently clear GAS in the URT mucosa, independent of GAS serotype.

To investigate whether 5CP conferred protection against systemic GAS infection, killing assays were performed using mouse whole blood. After 2 h of incubation of live GAS M1 cells with blood samples, the bacterial growth rate was significantly reduced in blood from 5CP-immunized mice, similar to blood from GAS infection-experienced mice (Fig. 3c). As expected, similar CFU reduction was also observed when GAS serotype M12 and M49 were used as target bacteria. To confirm these results, the recently developed GAS opsonophagocytic killing assay (OPKA) with differentiated HL-60 cells was used for detection of opsonic activity of anti-5CP serum from vaccinated mice (25, 26). There was a marked reduction in M1 CFU in the presence of sera from 5CP-immunized or infection-experienced mice. Similar CFU reductions were also observed when GAS M12 was incubated with HL-60 cells in the presence of sera from 5CP-immunized or infection-experienced mice (Fig. 3d).

Systemic protection was further tested *in vivo*. Immunized mice were i.n. challenged with a lethal dose of the M1 strain. CFU were recovered from the blood, spleen, and liver 24 h after the challenge (data not shown), indicating that the bacteria were disseminated through the body (27). Mouse survival was monitored daily over 14 days, and 82% to 90% of adjuvant- or PBS-immunized mice died within 6 days following challenge. In contrast, 95% of 5CP-immunized mice were alive (*P* < 0.0001) at the end of the experiment, comparable to survival rates in infection-experienced mice (Fig. 3e). Immunized mice were challenged with a lethal dose of a serotype M49 strain to evaluate systemic protection across serotypes. Compared with an 80% to 85% death rate in adjuvant- or PBS-immunized mice, only 30% of 5CP-immunized mice died within 6 days after challenge (*P* < 0.01) (Fig. 3f). These results demonstrate that 5CP immunization via the i.n. route can provide local and systemic protection against GAS, independent of serotype.

### Role of 5CP-induced immunity in protection against GAS skin infection.

**FIG 4:**
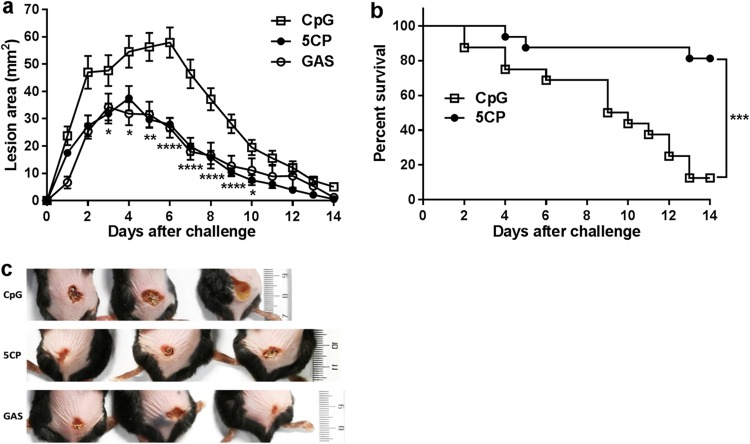
Role of 5CP-induced immunity in protection against skin infection by GAS. Two weeks after the last immunization, mice were s.c. challenged with 3 × 10^7^ CFU/mouse of GAS M1. (a) Skin lesion size was measured daily for 14 days. Data are from two independent experiments and presented as means ± SEM (*n* = 10 to 12). Statistical significance was determined by two-way ANOVA with multiple comparisons and indicated as follows: ****, *P* < 0.0001; **, *P* < 0.01; *, *P* < 0.05. (b) Survival curves following s.c. challenge with 1 × 10^8^ CFU/mouse of GAS M1. Data are from two independent experiments (*n* = 16). Curves were compared using the log rank test, and statistical significance was indicated as follows: ***, *P* < 0.001. (c) Representative images of mouse skin lesions at day 6 after s.c challenge with 3 × 10^7^ CFU/mouse of GAS M1 (*n* = 10 to 12). The scale to the right of the images is shown in centimeters.

GAS is one of the most common pathogens responsible for a wide variety of skin infections (28). A superficial non-life-threatening skin infection was employed to assess the efficacy of 5CP-induced immunity against skin infection. Mice were subcutaneously inoculated with 3.0 × 10^7^ CFU of GAS M1 (strain 90-226), and skin lesion size was measured daily for 14 days. After 3 or 4 days, lesion sizes reached maxima of 37.46 ± 4.53 and 34.28 ± 4.89 mm^2^ in 5CP-immunized and GAS infection-experienced mice, respectively, relative to 52.78 ± 4.08 mm^2^ in CpG-treated control mice. Further, while lesion size continually increased to a peak value (57.49 ± 6.39 mm^2^) in control mice on day 6, lesion size decreased to 27.90 ± 5.67 and 26.66 ± 8.40 mm^2^ in 5CP-immunized and infection-experienced mice, respectively (Fig. 4a and c). At the end of the experiment (day 14), lesions in the 5CP and GAS groups were 0.53 ± 0.23 mm^2^, with small central scabs and surrounding shrunken skin at the infection site. In contrast, the lesions were much larger in the CpG group, at 4.16 ± 0.657mm^2^ (Fig. 4a).

Severe skin and soft tissue infections caused by GAS often cause systemic shock and organ failure, leading to death (29). An invasive mouse model of disease was employed to assess the efficacy of 5CP against systemic infection via skin and soft tissue foci (14). Following subcutaneous challenge of immunized mice with 1 × 10^8^ CFU of GAS M1, the mortality rate was monitored over 14 days. By the end of the experiment, more than 80% of 5CP-immunized mice were alive. In contrast, less than 20% of CpG control mice survived (Fig. 4b). Taken together, the results from the superficial and invasive subcutaneous infection models indicate that the immune response to 5CP constrains skin infection and prevents death from invasive skin infection.

### 5CP induced long-lived plasma and Th17 cells and provided efficient long-term memory protection.

**FIG 5:**
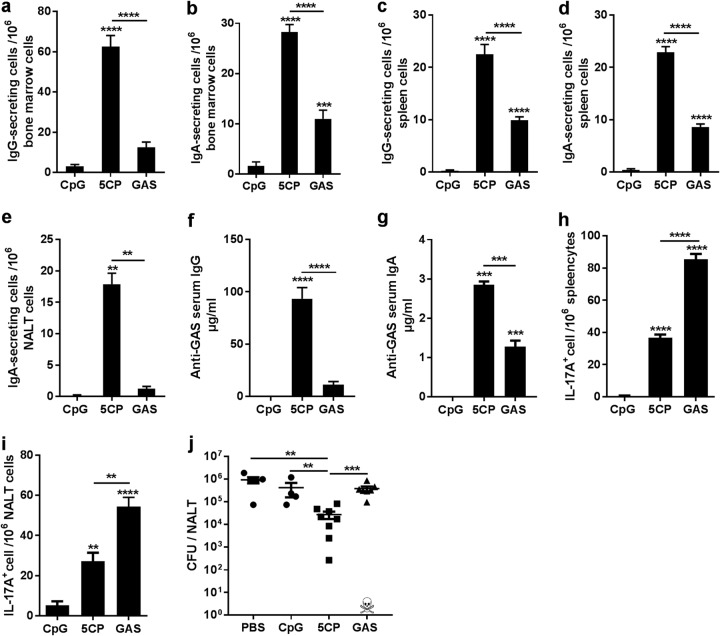
5CP induced long-lived plasma and Th17 cells and provided efficient long term-protection. (a to e) Six months after the last immunization, 5CP-specific antibody-secreting cells in the bone marrow (a and b), spleen (c and d), and NALT (e) were measured by ELISPOT assays. (f and g) GAS-specific serum IgG (f) and saliva IgA in mouth wash (g) were measured by ELISA. (h and i) Spleen (h) and NALT (i) cells were isolated from immunized mice 6 months after the last immunization and stimulated with HK GAS, and IL-17A-secreting cells were measured by ELISPOT assay. Data are presented as means plus SEM (*n* = 8). (j) Mice were intranasally challenged with 2 × 10^8^ GAS M1 (strain 90-226) 6 months after the last immunization. CFU in NALT were determined 24 h after challenge. Data from two independent experiments are presented as means ± SEM (*n* = 4 to 8). Statistical significance was determined by one-way ANOVA with Tukey’s posttest and indicated as follows: ****, *P* < 0.0001; ***, *P* < 0.001; **, *P* < 0.01.

Following vaccination or infection, antibody levels can persist for years to protect against reinfection. Long-lived plasma cells (LLPCs) are the primary source of these antibodies and primarily reside in the bone marrow (BM) (30). Similar to systemic immune responses, mucosal immunization induces LLPCs in the BM (31). To determine whether 5CP vaccination through the i.n. route induces LLPCs, antigen-specific LLPCs were assessed by ELISPOT assay 6 months after the last immunization. Compared with GAS infection-experienced mice, higher levels of 5CP-specific IgG^+^ and IgA^+^ LLPCs were found in the BM and spleen of 5CP-immunized mice (Fig. 5a, b, c, and d). IgA^+^-secreting LLPCs were also found in NALT (Fig. 5e). ELISAs revealed that long-lasting antibodies were maintained in the sera and URT mucosa and able to bind GAS cells (Fig. 5f and g), with higher levels of serum IgG and mucosal IgA in 5CP-immunized mice compared with infection-experienced mice. Additionally, long-lived Th17 cells that responded to whole GAS cells were detected in the spleens and NALT of 5CP-immunized and infection-experienced mice (Fig. 5h and i).

The efficacy of the long-term immunity in protecting against GAS was tested by i.n. challenge 6 months after the last dose of immunization. Assays of NALT in 5CP-immunized mice revealed <3.0 × 10^4^ CFU, while there were 1.0 × 10^6^ CFU in adjuvant immunized mice (Fig. 5j). Interestingly, CFU from GAS infection-experienced mice (one mouse in this group did not survive repeated low-dose infection before challenge) were not significantly reduced compared with adjuvant-immunized mice. These results indicate that i.n. immunization with 5CP can induce long-lasting protective immunity against GAS infection.

### The Th17 response induced by 5CP was resolved promptly.

**FIG 6:**
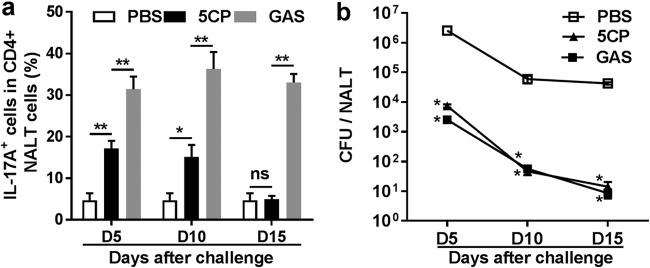
Th17 response induced by 5CP was resolved promptly. Immunized mice were challenged and sacrificed on the indicated days (days 5, 10, and 15). (a) IL-17A^+^ CD4^+^ cells in NALT on the indicated days were determined by flow cytometry. (b) CFU recovered from NALT. Data are presented as means ± SEM from two independent experiments (*n* = 8). Statistical significance was determined by two-tailed Student’s *t* test and indicated as follows: **, *P* < 0.01; *, *P* < 0.05; ns, not significant.

The Th17 response is important for efficient clearance of GAS; however, sustained GAS-specific Th17 responses are implicated in GAS-related pathological reactions and autoimmune disorders (32). Therefore, for GAS vaccines, a controlled Th17 response is crucial. We previously reported that repeated GAS infection induced a strong Th17 response, which was sustained at a high level after bacteria were cleared. In contrast, the Th17 response to a combined GAS subunit vaccine resolved in a timely fashion (6). To determine the duration of the Th17 response to 5CP, immunized mice were i.n. challenged with GAS, and Th17 cells in NALT were examined over 15 days after challenge. Compared with naive mice, high levels of activated Th17 cells were maintained in GAS infection-experienced mice at least 15 days after challenge, and even the challenge bacteria were cleared (Fig. 6a and b). In contrast, Th17 cells in 5CP-immunized mice were moderately activated at day 5 and maintained these levels at day 10, returning to baseline by day 15 (Fig. 6a), with a similar kinetics of bacterial clearance to that of infection-experienced mice (Fig. 6b). These results indicate that 5CP induces protective immunity as efficiently as live GAS, with a promptly resolved Th17 response.

## DISCUSSION

As a successful pathogen, GAS expresses a range of virulence factors for colonization, invasion, and dissemination in tissues to ensure infection (33, 34). Various human disease manifestations are caused by this pathogen, from mild skin and throat infections to systemic life-threatening conditions, suggesting that multiple components in the virulence arsenal of the bacteria are involved in the diverse diseases; however, candidate subunit vaccines that show protection in multiple murine models simulating human disease caused by GAS have not been reported. In this study, five highly conserved GAS components were selected based on their roles in the critical events of pathogenesis and combined as a multiple subunit vaccine. Our results demonstrate that immunization of mice through the i.n. route with 5CP induces both local and systemic immune responses across serotypes and provides protection against GAS in mucosal, systemic, and skin infection models.

Besides the M proteins, several highly conserved non-M components of GAS can induce protection in murine infection models when used individually and are antigenic in humans (5, 6, 11, 16, 17, 19, 35); however, single-antigen vaccines may provide protection in one challenge model, but not in another (36, 37). Protection against different types of infection may require a distinct set of immune responses. Thus, inclusion of multiple virulence factors involved in various GAS pathogenic mechanisms in a vaccine would provide protection across various GAS diseases.

Recent studies of vaccines have shown that multicomponent formulations are protective (6, 11, 12, 14). More recently, an experimental GAS vaccine (Combo5) that contains five components was evaluated in a nonhuman primate (NHP) model of GAS pharyngitis, and the results showed a reduction in pharyngitis and tonsillitis in immunized NHPs relative to controls (38). The consistent efficacy of these vaccines indicates that the included components and the strategy of combining multiple factors result in promising efficacy. In this study, the components included in 5CP formulations target the critical pathogenic mechanisms, such as adherence (SpyAD) (39), phagocytosis (SCPA) (40), cytolysis (SLO) (41), chemotaxis (SpyCEP) (18), and other processes carried out by SrtA-anchored additional membrane proteins that contain the LPXTG motif (42). We previously reported that the immunity induced by the two-component vaccine, SrtA/SCPA, reduces mucosal colonization but not lethality. In this study, 5CP, which contains three more components, protected mice from mucosal colonization and lethality as efficiently as whole GAS cells, indicating that the immunity induced by 5CP is more competent to prevent GAS pathogenesis and suggesting that combining more important virulence factors in a vaccine can increase protection efficacy covering different GAS diseases. Human antibodies directed to each 5CP component indicate that 5CP is antigenic in humans. Whether 5CP can protect humans from various GAS diseases requires testing. In addition, optimization of the 5CP formulation could further improve the efficacy of the vaccine.

Similar to previously reported studies (4), parenteral immunization with 5CP failed to reduce mucosal GAS colonization, despite induction of high levels of serum IgG (see Fig. S2 in the supplemental material). In contrast, in this study, i.n. immunization with 5CP protects mucosal infection efficiently. Because the URT mucosa is the reservoir and entry port for GAS, reducing GAS colonization at mucosal sites would limit the chances of dissemination and contribute to protection against systemic infection and lethality. In addition, i.n. immunization is associated with long-term protection, although the underlying mechanisms are incompletely understood. Immunity against the URT pathogen Bordetella pertussis induced by natural infection is longer lasting than that induced by parenteral immunization (43), suggesting that it is logical to use the nasal route to vaccinate with the aim of generating enduring protective immunity.

10.1128/mBio.02600-19.2 FIG S2 Subcutaneous immunization with 5CP did not provide protection against group A streptococcus (GAS) mucosal infection. Mice were intranasally (i.n.) or subcutaneously (s.c.) immunized with 5CP or CpG as described in Materials and Methods. IR, immunization route. (a) Two weeks after the last immunization, mice were i.n. challenged with GAS M1. CFU in NALT were determined 24 h after challenge. Data are presented as means ± SEM (*n* = 5). (b to d) Two weeks after the last immunization, antibodies in serum and mouthwash samples were measured by ELISA. Levels of serum IgG (b) and IgA in mouth wash directed to 5CP (c). Levels of IgA in mouth wash directed to GAS (d). Data are presented as means ± SD (*n* = 5). Statistical significance was determined by two-tailed Student’s *t* test and indicated as follows: **, *P* < 0.01; ns, not significant. Download FIG S2, PDF file, 0.2 MB. Copyright © 2019 Bi et al. 2019 Bi et al. This content is distributed under the terms of the Creative Commons Attribution 4.0 International license.

GAS-specific Th17 cells are implicated in GAS-related autoimmune disorders (7). Association of the Th17 response to autoimmune disease was recently demonstrated in a mouse model (32). As Th17 cells are mainly produced through the i.n. route, such as natural GAS infection, resolution of the Th17 response after GAS clearance is critical for vaccine safety. Consistent with our previous study of the SrtA/SCPA vaccine (6), the Th17 response to 5CP resolved more rapidly than that induced by GAS and responded to GAS challenge as significantly as that generated by whole GAS cells, suggesting that the Th17 response to 5CP is competent, with less potential to trigger autoimmunity.

Overall, the results of this study indicate that the 5CP formulation and immunization through the i.n. route are necessary for protective efficacy across GAS serotypes and challenge models. The GAS vaccine strategy used in this study generates effective, broad, and enduring responses.

## MATERIALS AND METHODS

### Ethics statement.

All animal procedures in this study were performed in strict accordance with the recommendations in the Guide for the Care and Use of Laboratory Animals of the Institute of Microbiology, Chinese Academy of Sciences (IMCAS) Ethics Committee. The protocols were approved by the Committee on the Ethics of Animal Experiments of IMCAS (permit APIMCA2019030). Mice were bred under specific-pathogen-free conditions in a laboratory animal facility at IMCAS. All animal experiments were conducted under isoflurane anesthesia, and all efforts were made to minimize suffering. The collection and use of samples from patients were approved by the Ethics Committee of Beijing Children’s Hospital and Beijing Red Cross Blood Center.

### Bacterial strains.

GAS serotype M1 (strain 90-226) was obtained from the University of Minnesota. Serotypes M12 and M49 were clinical isolates obtained from Beijing Children’s Hospital. All strains were maintained on sheep blood agar and grown in THB-Neo broth at 37°C in 5% CO_2_. Overnight cultures (optical density at 560 nm [OD_560_], approximately 1.1) were washed with and resuspended in PBS and then used for infection or challenge. CFU were verified by plating on blood agar plates.

### Cloning and purification of vaccine antigens.

DNA sequences for the selected antigens were amplified from GAS serotype M1 (strain 90-226). Recombinant SrtA and SCPA were cloned into the pET28a vector as previously described (5, 6). Recombinant SpyAD (amino acids 37 to 849) was cloned into the pET30a vector (11). CEP-5 (amino acids 35 to 587) was a portion of the group A streptococcus cell envelope proteinase (SpyCEP) that included the D151A mutation to remove the bioactivity and was cloned into the pET30a vector (17). SLO (amino acids 32 to 571), with P427L and W535F site mutations, was cloned into the pET-28a vector (11, 19). All constructs were transformed into Escherichia coli BL21(DE3) for expression. Recombinant proteins were purified as previously described (5). Lipopolysaccharide was removed from the purified proteins to <0.1 endotoxin units (EU)/μg recombinant protein, using a ToxinEraser endotoxin removal kit (Genscript, USA) following the manufacturer’s protocol.

### Immunization and challenge of mice.

Female BALB/c mice (aged 4 to 6 weeks) were purchased from Vital River Laboratory Animal Technology, whose colonies were all introduced from Charles River Laboratories. Mice were anesthetized with an isoflurane/oxygen mixture and i.n. inoculated with 50 μg of 5CP (10 μg of each recombinant protein) with 10 μg of CpG-oligodeoxynucleotides (CpG-OND 1826; Generay Biotechnology, China) as adjuvant, in a 10-μl volume/mouse (5 μl per nostril). For subcutaneous (s.c.) immunization, 50 μg of 5CP, with 10 μg CpG-OND 1826 as adjuvant, in 50-μl volume/mouse was s.c. injected into the upper back. A low dose (0.5 × 10^8^ to 1 × 10^8^/mouse) of live GAS M1 (strain 90-226) was used for infection. Control mice were administered PBS or 10 μg of CpG-OND in PBS. Mice were immunized or infected three times at 1-week intervals. For the mucosal challenge model, mice were i.n. challenged with GAS serotype M1 (strain 90-226) or serotype M12 at a sublethal dose of 2.0 × 10^8^/mouse, 2 weeks after the last immunization. CFU in NALT were determined 24 h postchallenge, as previously described (5). For the systemic infection model, mice were challenged i.n. with a lethal dose (3 × 10^8^/mouse) of GAS serotype M1 (strain 90-226) or M49, and weight loss and survival were monitored twice per day for 14 days. Mice with weight loss of 25% of the starting body weight were euthanized and recorded as dead. Mortality was an anticipated outcome and approved by the animal ethics committee. Surviving mice were humanely euthanized at the end of the experiment. For the skin abscess model, i.n. immunized mice were anesthetized and s.c. challenged on the shaved lower back with 3 × 10^7^ CFU/mouse of GAS M1 (strain 90-226; 50 μl). The size of dermonecrotic lesions was measured daily. For the invasive subcutaneous infection mouse model, immunized mice were s.c. challenged with 1 × 10^8^ CFU/mouse of GAS M1 (strain 90-226; 50 μl), and survival was monitored for 14 days.

### Human serum samples and human peripheral mononuclear blood cells.

Serum samples from children aged 5 to 15 years were obtained from Beijing Children’s Hospital (Beijing, China), and human PBMCs were obtained from Beijing Red Cross Blood Center (Beijing, China).

### ELISA for antibodies and IL-17A.

GAS- and antigen-specific antibodies were measured by ELISA as previously described (44). Plates were coated with 3 × 10^7^/ml of heat-killed (HK) GAS M1 serotype (strain 90-226) or 5 μg/ml of single recombinant antigen in PBS and incubated overnight at 4°C. Aliquots (100 μl) of each serum sample were added to each well of plates and incubated at 37°C for 2 h. Horseradish peroxidase (HRP)-conjugated goat anti-mouse IgG or goat anti-mouse IgA were used to detect mouse antibodies. HRP-conjugated goat anti-human IgG was used for detection of human serum IgG. IL-17A levels in the supernatants of NALT homogenate were measured using ELISA kits (eBioscience, USA) as previously described (44).

### Whole-blood killing assay.

Mouse whole-blood killing assays were conducted as previously described (45) with minor modifications. Mouse blood was freshly isolated from immunized mice and mixed with heparin. Live GAS cells were diluted to the desired concentration in PBS, and 20 μl of bacterial suspension containing 1 × 10^3^ CFU was added to 80 μl of mouse blood in 1.5-ml siliconized tubes. Tubes were then placed in a rotating rack and incubated at 37°C for 2 h, and reaction mixtures were subsequently serially diluted and plated on blood agar plates for CFU determination. The bacterial growth rate was calculated as mean CFU of test serum tube/mean CFU of input inoculum.

### HL-60 opsonophagocytic killing assay.

HL-60 opsonophagocytic killing assay (OPKA) was conducted as previously described (25). Briefly, HL-60 cells were differentiated into neutrophil-like cells by culture in RPMI medium (Invitrogen, Life Technologies Ltd., CA, USA) containing 0.8% *N*,*N*-dimethylformamide (DMF) for 5 days. Differentiated HL-60 cells were assessed by flow cytometry to confirm that ≥55% and ≤20% of the total cells were CD35^+^ and CD71^+^ (Biolegend, USA), respectively. OPKA reactions were performed in round-bottomed 96-well plates with heat-inactivated (56°C for 30 min) serum from immunized mice, 1 × 10^3^ CFU of GAS in opsonization buffer, 4 × 10^5^ differentiated HL-60 cells, and 5% baby rabbit complement (Pel-Freez, AR, USA). Control reactions were conducted using heat-inactivated serum from naive mice without differentiated HL-60 cells. Reaction mixtures were incubated for 1.5 h at 37°C with 200 rpm shaking. CFU were determined by plating serial 10-fold dilutions on blood agar plates. The bacterial growth rate was calculated as mean CFU of test serum well/mean CFU of control well.

### T cell analysis by flow cytometry and ELISPOT assays of T and B cells.

Cellular staining and flow cytometry analyses of T cells were conducted as previously described (6). Samples were analyzed using a FACS Aria II flow cytometer (BD Biosciences) and FlowJo software (Tree Star). Long-lived GAS-specific Th17 cells were examined using a T cell ELISPOT kit (Mabtech, Minneapolis, MN, USA), according to the manufacturer’s instructions. Briefly, single-cell suspensions of BM, spleen, NALT, or human PBMCs (4 × 10^5^ BM or splenic cells, 2 × 10^5^ NALT cells, or 2 × 10^5^ human PBMCs, per well) were seeded in 96-well plates. Cells were cocultured with HK GAS (multiplicity of infection of 10) or each antigen from 5CP individually (20 μg/ml) for 24 h. Antigen-specific LLPCs in the BM, NALT, and spleen were quantified by ELISPOT assays using specific antibody-secreting cells as described previously (46). All plates were developed using procedures established for 3-amino-9-ethylcarbazole (AEC) (Millipore). Spots were enumerated using an ImmunoSpot Analyzer (Cellular Technology Ltd., OH, USA).

### Statistical analysis.

Statistical analyses were performed using GraphPad Prism software (version 7.0). The CFU were log_10_ transformed before the statistical analysis. When two groups were compared, the CFU were analyzed by two-tailed unpaired Mann-Whitney *U* nonparametric *t* test, and other variables were compared by two-tailed Student’s *t* test. One-way analysis of variance (ANOVA) with Tukey’s posttest was used to analyze the statistical significance of differences among more than two groups. Survival curve was analyzed using the log rank test. When two independent variables were compared, data were analyzed by two-way ANOVA with multiple-comparison test as stated. A *P* value of <0.05 was considered significant.
