# Supplementary figures and images for: A Multicomponent Vaccine Provides Immunity against Local and Systemic Infections by Group A Streptococcus across Serotypes
Source: mBio. 2019 Nov 26;10(6):e02600-19. doi: 10.1128/mBio.02600-19 (PMC6879722; doi:10.1128/mBio.02600-19)

# Figure S1

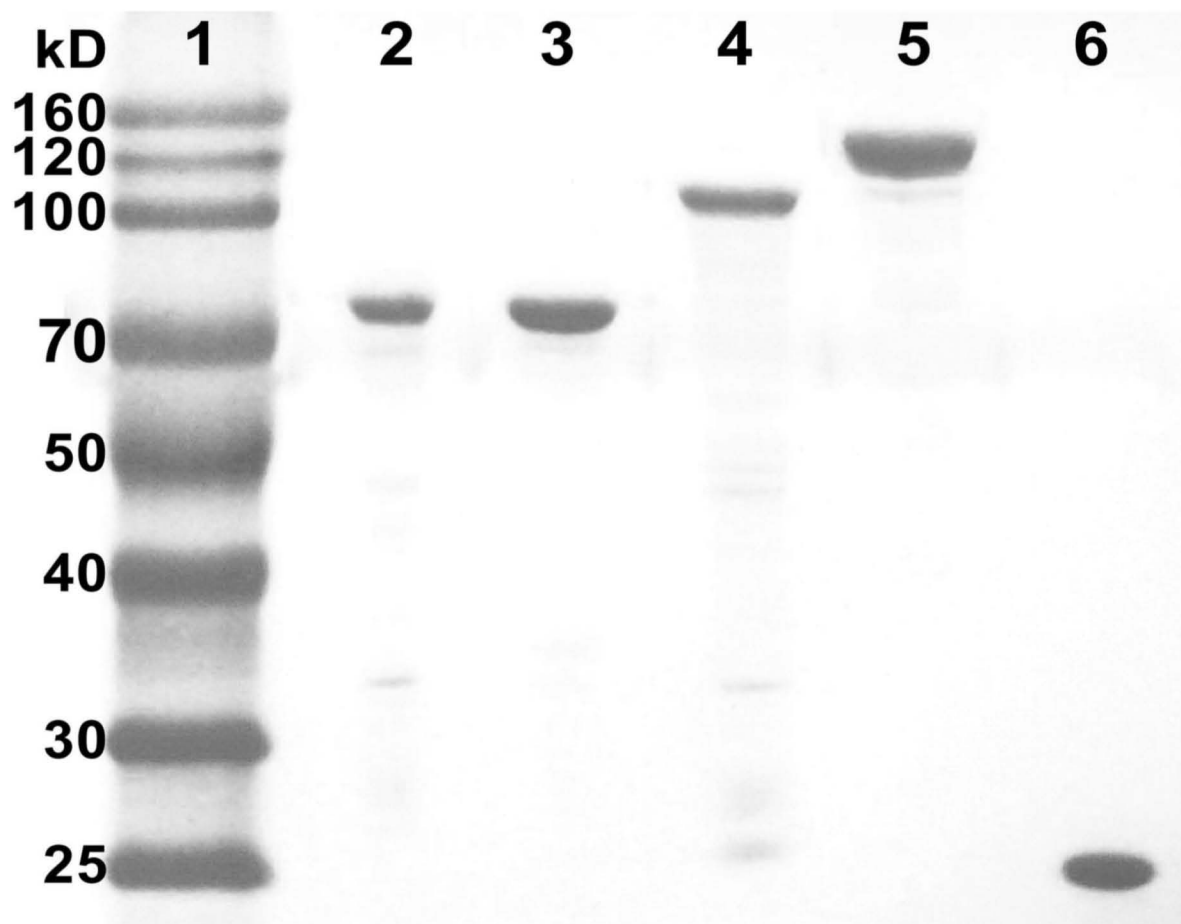

Supplement: FIG S1 [file mBio.02600-19-sf001.pdf]

**Figure S2**

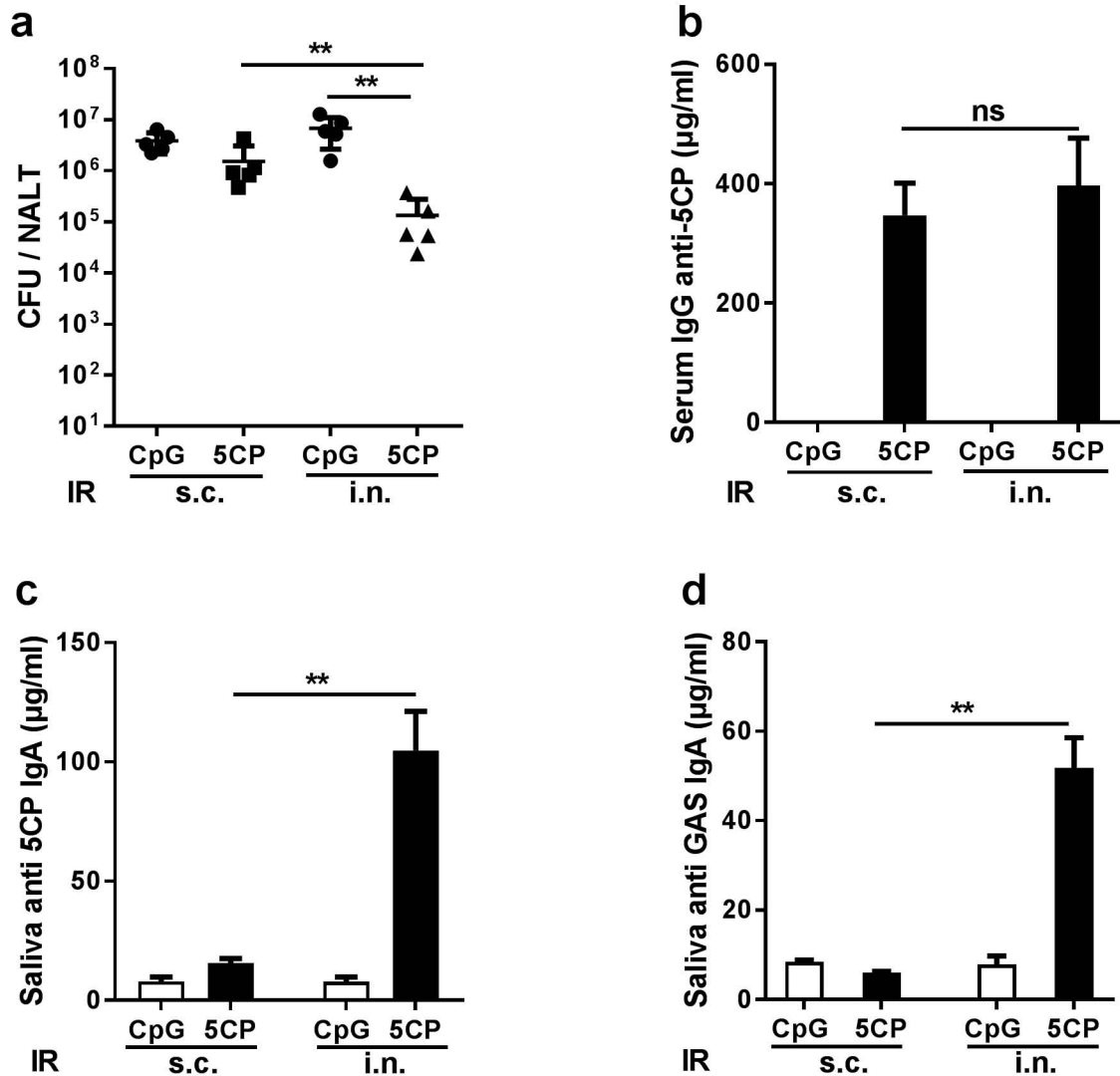

Supplement: FIG S2 [file mBio.02600-19-sf002.pdf]
